# Education Research: Trends of Neurology Clerkships in the United States Amidst a Global Pandemic

**DOI:** 10.1212/NE9.0000000000200169

**Published:** 2024-11-06

**Authors:** Stephen VanHaerents, Doris Kung, Erin Furr-Stimming, Jeremy K. Cutsforth-Gregory, Joshua Weaver, Carolyn M. Cahill, Tasha Ostendorf, Christopher M. Keran, as the AAN Undergraduate Education Subcommittee

**Affiliations:** From the Department of Neurology Feinberg School of Medicine (S.V.), Northwestern University, Chicago, IL; Baylor College of Medicine (D.K.), Houston, TX; University of Texas Health Science Center at Houston (E.F.S.), McGovern Medical School; Mayo Clinic (J.K.C.-G.), Rochester, MN; Weill Cornell Medical College (J.W.), New York, NY; American Academy of Neurology (C.M.C., T.O., C.M.K.), Minneapolis, MN.

## Abstract

**Background and Objectives:**

To report a 2022 survey of US medical school neurology clerkship directors (CDs) and to compare the results with those of similar surveys conducted in 2005, 2012, and 2017.

**Methods:**

An American Academy of Neurology (AAN) Consortium of Neurology Clerkship Directors (CNCD) workgroup developed the survey sent to all neurology CDs listed in the AAN CNCD database. Comparisons were made with 2005, 2012, and 2017 surveys.

**Results:**

The response rate was 72 (47%) of 152 CDs. The number of respondents reporting a required neurology clerkship has not significantly changed over the past 17 years (93%, 93%, 94%, and 96% for 2005, 2012, 2017, and 2022, respectively, *p* = 0.848), but the timing of the clerkship has shifted. The proportion of clerkships that occur exclusively in students' third year has fluctuated around 50% since 2012 (56% in 2012, 51% in 2017, and 58% in 2022). Those taken only in the fourth year have decreased (from 45.6% in 2005, to 12% in 2012, 9% in 2017, and 3% in 2022 *p* < 0.001). Three-fourths of respondents (n = 54 of 72) report a 4-week clerkship duration, the same as past years (75% in 2017 and 75% in 2012). During the initial stages of the coronavirus disease 2019 (COVID-19) pandemic (March 2020–July 2021), students in the neurology clerkship spent an average of 6 hours in online learning compared with 3 hours currently (*p* < 0.001). Respondents received an average of 27% of a full-time equivalent protected time. 87% indicated that they are very or somewhat satisfied in their role, but most (60%, n = 38/63) indicated that they have experienced burnout at some point within their career as a neurology CD.

**Discussion:**

Neurology clerkships remained resilient in the United States through the initial stages of the COVID-19 pandemic with demonstrated consistency in structure, curriculum, and administration. There has been a continued increase in the requirement of the clerkship as a 4-week clinical experience. The needs persist for more clerkship coordinator support and more protected time for neurology CDs. Despite high satisfaction, most of the respondents have experienced burnout for a variety of reasons, including lack of protected time and competing obligations.

## Introduction

The goal of a neurology clerkship is to provide essential clinical training to medical students to ensure that all graduates have the medical knowledge and clinical skills to identify and care for patients with common and/or emergent neurologic symptoms and disorders, regardless of their selected specialty. A neurology clerkship also provides opportunities for medical students to explore clinical neurology and for educators to identify and begin to train the next generation of neurologists.^1^ This is particularly important because the current supply and demand gap in the country's neurology workforce is expected to continue in the near future.^2^ In addition, clerkship directors (CDs) are responsible for adapting their programs to respond to the expanding science of neurology and to regulatory changes to curriculum delivery seen most prominently during the initial stages of the coronavirus disease 2019 (COVID-19) pandemic. The Consortium of Neurology Clerkship Directors (CNCD) of the American Academy of Neurology (AAN) performed previous surveys of neurology CDs in the United States in 2005, 2012, and 2017. The 2017 survey reported changes between 2005 and 2012 that included shifting the neurology clerkship to earlier in the medical school curriculum, increasing CD salary support, rising CD satisfaction, and a lack of racial diversity among CDs.^3^ Significant changes to medical education have occurred since 2017, most notably with the COVID-19 pandemic, and thus, the AAN sought to update these data and ask new questions to address changes to clerkships and undergraduate medical education training.

## Methods

### Standard Protocol Approvals

This study (HSC-MS-22-0833—Neurology Clerkship Director Survey 2022) was reviewed by the institutional review board (IRB) of the University of Texas and granted IRB exempt status according to 45 CFR 46.104 (d).

### Instrument

Questions were developed by members of the AAN staff, the AAN CNCD, and AAN Undergraduate Education Subcommittee. The instrument was reviewed and approved by the Member Research Subcommittee. The 2022 survey used the 2017 instrument as a guide, with the addition of new questions on telemedicine exposure, curriculum changes due to COVID-19, and trainee assessments (eAppendix 1).

### Population

A roster containing all US neurology CDs was pulled from the AAN's internal database on November 2, 2022. The CD roster is updated whenever a program contacts the AAN to indicate a change in leadership. The list contained 152 CDs with valid email addresses.

### Data Collection

The survey was conducted entirely online from November 2 through December 9, 2022. Two reminder emails were sent to nonrespondents, one on November 11 and another on November 16. Messages were also posted in the CNCD Synapse group, encouraging members to complete the survey. CNCD survey workgroup members emailed nonrespondents, encouraging them to participate.

### Response Rate and Representativeness

The survey yielded a 47% response rate (72/152).

### Analysis

Basic descriptive and summary statistics were calculated using SPSS version 26 and are included in the frequencies section. Write-in responses with over 20 comments were coded by theme. Pearson χ^2^ testing was performed to assess differences among the years.

### Data Availability

Any data not published in the article will be shared by request.

## Results

The response rate for the 2022 survey was 47% (72 of 152 programs), lower than for the 2017 survey, which was 63% (92 of 146 programs), and the 2012 and 2005 surveys (73.1% in 2012 and 75% in 2005). The number of CDs invited to the survey numerically increased substantially from 2005 (109) to 2022 (152). Full statistics and original survey questions can be found in the supplemental data.

### Structure of the Neurology Clerkship

#### Timing

The percentage of respondents reporting a required neurology clerkship has not significantly changed over the past 17 years (93%, 93%, 94%, and 96% for 2005, 2012, 2017, and 2022, respectively, *p* = 0.848), and the timing of the clerkship has shifted earlier in the curriculum. As given in Table 1, the proportion of clerkships taken exclusively in the third year has hovered just above 50% since 2012 (56% in 2012, 51% in 2017, and 58% in 2022). In 2005, 45% of respondents reported their clerkship in the third year. Those taken only in the fourth year have decreased (from 22.5% in 2005 to 3% in 2022, *p* < 0.001). In addition, the proportion of respondents reporting that the clerkship is taken in “other” years was lower in 2005 (1%) compared with 12% in 2012, 16% in 2017, and 20% in 2022 (*p* < 0.001). This “other” category includes longitudinal clerkships and those taken in the second year. 75% of respondents reported a 4-week clerkship duration, which is unchanged as in past years (85.4% in 2005, 75% in 2012, and 75% in 2017, *p* = 0.314).

**Table 1:** Characteristics of Neurology Clerkships

	2005^a^ (n = 82)	2012^b^ (n = 98)	2017^c^ (n = 92)	2022^d^ (n = 72)	Column comparison^1^
Q1 Neurology clerkship required? Yes (%)	92.7	92.9	93.5	95.8	*p* = 0.848
Q3 Clerkship duration: 2 wk (%)	4.9	9.2	5.4	5.6	*p* = 0.314
Q3 Clerkship duration: 3 wk (%)	7.3	11.2	10.9	6.9	
Q3 Clerkship duration: 4 wk (%)	85.4	74.5	75.0	75.0	
Q3 Clerkship duration: others (%)	2.4	5.1	8.7	12.5	
Q4 Timing of the clerkship: 3rd year (%)	45.0	56.1	51.1	58.0	*p* < 0.001
Q4 Timing of the clerkship: 4th year (%)	22.5^d^	12.2	8.7	2.9	
Q4 Timing of the clerkship: 3rd or 4th year (%)	31.3	19.4	23.9	18.8	
Q4 Timing of the clerkship: other (%)	1.3^b,c,d^	12.2	16.3	20.3	
Q33 Importance of teaching in promotion: not at all (%)	12.8	5.2	7.9	4.6	*p* < 0.001
Q33 Importance of teaching in promotion: somewhat (%)	71.8^c,d^	62.5^d^	49.4	35.4	
Q33 Importance of teaching in promotion: very (%)	15.4	25.0	24.7	38.5^a^	
Q33 Importance of teaching in promotion: extremely (%)	0.0	7.3	18.0	21.5^b^	
Q49 Administration support FTE: 0–0.25 (%)	51.9	46.9	35.0	27.4	*p* < 0.001
Q49 Administration support FTE: 0.26–0.50 (%)	36.4^c,d^	24.0^d^	11.3	14.5	
Q49 Administration support FTE: 0.51–0.75 (%)	3.9	14.6	35.0^a,b,d^	24.2	
Q49 Administration support FTE: 0.76–1.0 (%)	7.8	14.6	18.8^c^	33.9^a^	
Q45 CD protected time FTE: 0%–5% (%)	37.7	14.6	9.3	4.8	N/A^2^
Q45 CD protected time FTE: 6%–15% (%)	24.7	27.1	15.1	22.6	
Q45 CD protected time FTE: 16%–25% (%)	29.9	38.5	43.0	29.0	
Q45 CD protected time FTE: 26%–50% (%)	6.5	18.8	31.4	41.9	
Q45 CD protected time FTE: >50% (%)	1.3	1.0	1.2	1.6	
Q51 Faculty rank: assistant professor (%)	32.1	33.3	29.5	38.5	*p* = 0.961
Q51 Faculty rank: associate professor (%)	39.5	37.5	43.2	40.0	
Q51 Faculty rank: full professor (%)	24.7	27.1	25.0	18.5	
Q51 Faculty rank: others (%)	3.7	2.1	2.3	3.1	
Q53 Clinical educator track: Yes (%)	N/A	48.4	57.5	57.8	*p* = 0.593
Q54 Years in role: 0–6 (%)	67.1	57.3	47.7	57.1	*p* = 0.167
Q54 Years in role: 7–12 (%)	24.4	27.1	29.1	23.8	
Q54 Years in role: >12 (%)	8.5	15.6	23.3	19.0	
Q61 Member of the CNCD: yes (%)	66.7	76.0	83.1	84.4	*p* = 0.036
Q62 Satisfaction with role: very satisfied (%)	N/A	34.4	48.9	53.1	*p* = 0.028^3^
Q62 Satisfaction with role: somewhat satisfied (%)	N/A	51.0	46.6	34.4	
Q62 Satisfaction with role: neutral/dissatisfied (%)	N/A	14.6	4.5	12.5	
Q63 Experienced burnout: yes, frequently (%)	N/A	11.5	14.8	15.9	*p* = 0.812
Q63 Experienced burnout: yes, but infrequently (%)	N/A	45.8	50.0	44.4	
Q63 Experienced burnout: no (%)	N/A	42.7	35.2	39.7	
Q64 Relinquish role in the next 12 mo: yes (%)	N/A	34.7	30.7	32.8	*p* = 0.843
Q65 Relinquish role: “hassle factor” (%)	N/A	40.6	52.0	45.0	N/A
Q65 Relinquish role: lack chair support (%)	N/A	18.8	32.0	35.0	
Q65 Relinquish role: lack admin support (%)	N/A	37.5	20.0	35.0	
Q65 Relinquish role: lack protected time (%)	N/A	53.1	44.0	50.0	
Q65 Relinquish role: competing obligations (%)	N/A	84.4	60.0	55.0	
Q65 Relinquish role: transition role to junior faculty (%)	N/A	N/A	44.0	45.0	

Abbreviations: CD = clerkship director; CNCD = Consortium of Neurology Clerkship Directors; FTE = full-time equivalent.

Superscript letters indicate the Pearson χ^2^ test (*p* < 0.001).

1 Pearson χ^2^ test.

2 χ^2^ is invalid because low cell counts.

3 No significant pairwise comparisons.

#### Curricular and Clinical Experiences

76% of respondents use the CNCD-published neurology clerkship core curriculum.^3^ Those who are not using the core curriculum use a different set of objectives (75%), are unaware of the publication (31%), or have other reasons (6%). On average, medical students in the neurology clerkship work up 5 new inpatients per week (range of 0–18) and 6 new outpatients per week (range of 0–40). Although the estimated number of students reported by CDs performing a lumbar puncture (LP) during the clerkship remains low, 59% of respondents offer LP simulation training. Respondents report that multiple methods are used to ensure that all students are observed by a faculty member taking a neurologic history and performing a neurologic examination during the clerkship: 64% use live patient interactions with a standardized form; 32% use live patient interactions without a standardized form; 30% use observed interactions with standardized patients; and 14% use other methods including feedback cards, direct CD interactions, and simulations. Sixty-two percent of respondents recommend a particular textbook or textbooks for suggested study during the neurology clerkship (Table 2). A variety of online resources are recommended by respondents. 63% of respondents use lecture videos. Fifty-three percent recommend eBooks. The other most common online resources include AAN Medical Student Self-Assessment Exam (47%), neurology-related apps (40%), online case simulation (33%), AAN NeuroBytes videos (33%), podcasts (30%), AAN Neurology Question of the Day app (12%), book exchange (5%), and others (28%), which included *Continuum: Lifelong Learning in Neurology*, websites, and question banks. To meet Liaison Committee on Medical Education (LCME) required clinical encounters in the neurology clerkship, 88% of respondents have students see a mandated number of patients from certain broad diagnostic clusters. Other strategies CD respondents use include small group discussions about a clinical entity (53%), lectures about an entity (50%), videos of patients with certain disorders (30%), simulated patients for certain diagnoses (20%), and others (9%) including using custom case-based resources, self-study, and symptom-based approaches.

**Table 2:** Recommended Text Books for Use During the Neurology Clerkship

Text book	No. of respondents recommending texts
Simon et al. *Clinical Neurology*^4^	12
Toy et al. *Case Files: Neurology*^5^	7
Drislane. *Blueprints Neurology*^6^	6
Gelb. *Introduction to Clinical Neurology*^7^	4
Kaplan and Milligan. *The Neurology Video Primer*^8^	3
Ropper et al. *Adams and Victor's Principles of Neurology*^9^	3
Anschel and DiCapua. *Neurology: PreTest Self-Assessment and Review*^10^	2
Berkowitz. *Clinical Neurology & Neuroanatomy: A Localization-Based Approach*^11^	2
Blumenfeld. *Neuroanatomy through Clinical Cases*^12^	2
Harrison et al. *Harrison's Neurology in Clinical Medicine*^13^	2
Johnson. *Raven Neurology Review: Clinical Neurology for Medical Students*^14^	2
Louis et al. *Merritt's Neurology*^15^	2
Biller. *Practical Neurology*^16^	1
Cucchiara and Price. *Decision-Making in Adult Neurology*^17^	1

### Evaluation Methods for the Neurology Clerkship

Direct observation of students by faculty and house officers is the largest weighted component of the final grade (mean 43%) reported by respondents. The second highest component is the National Board of Medical Examiners clinical neurology participant examination or “shelf exam” (mean 21%, range 0%–50%). Direct observation and the shelf exam were also the highest weighted components of the grading rubric in 2017. The shelf exam is used by most (83%) of clerkships responding. Reasons for not using it include preference for another form of assessment of medical knowledge (55%), preference for an institution-specific written examination (36%), concern that students' focus on studying for examination will detract from the clerkship (36%), preference for clinical assessment by observers (27%), or cost (27%). Of those who do not use the shelf exam, none (0%) responded that it is inconvenient to use or that they prefer an objective structured clinical examination or simulated patient assessment. Other methods used for grading and the mean percentage contribution to the final grade include the following: attendance and participation (9%), objective structured clinical examination (6%), written essay examination or case discussion (3%), other multiple-choice question examinations (3%), short-form quizzes (2%), and structured oral examination (observing a history and physical or discussing vignettes; 2%).

The proportion of respondents in 2022 who use a normative scale for grades (i.e., top 20% = honors) has remain unchanged over the past 5 years (30% in 2022 [n = 19/64] vs 42% in 2017 [n = 36/86]), in favor of competency-based grading (*p* = 0.126). One hundred percent of respondents stated that attending neurologists assess student performance (contributing to grades) during the neurology clerkship. Other contributors to the grade include residents (91%), fellows (61%), interns (28%), and nonphysician staff (e.g., advanced practice providers and nurses; 11%). 97% of respondents use standardized evaluation forms to ensure that students are assessed consistently in the neurology clerkship. Other methods for ensuring consistency in assessment are formal training for individuals who assess students (55%), minimum number of hours observing the student (27%), and others (1%) including teaching guides for evaluators. Respondents most often obtain feedback about the clerkship from students through written or online forms (98%). Additional methods include group interviews with students (38%), individual interviews with students (36%), and informal discussion/committee meetings (34%). Information from faculty and house officers about the program is most often obtained from informal discussions (85%), group interviews (36%), educational retreats (26%), written or online evaluation forms (25%), and individual interviews with faculty and house officers (20%).

### Curricular Changes During Initial Stages of the COVID-19 Pandemic (March 2020–July 2021)

During the initial COVID-19 “surge,” which the survey defined as March 2020–July 2021, students in the neurology clerkship spent an average of 6 hours in online learning, 3 hours in large group lectures, and 3 hours in small group teaching and learning activities per week. At the time of the 2022 survey (i.e., “after surge”), neurology clerkship students spend approximately half as much time in online learning (3 hours) than during the COVID-19 surge (6 hours) (*p* < 0.001) and more time in small group lectures (4 hours after COVID-19 surge vs 3 hours during COVID-19 surge, *p* = 0.002) but remain relatively unchanged in time devoted to large group lectures (3 hours, *p* = 0.631). Respondent grading methods are given in Table 3, with 42% using “Pass, Fail” during the COVID-19 “surge” that is larger than in all other years in this analysis (*p* < 0.001). In line with an increasing number of medical schools considering across-the-board “Pass, Fail” models of grading and competency-based curricula,^18^ the proportion of neurology clerkships using “Pass, Fail” has increased over time from 2.1% in 2012 (n = 2/95) to 16% in 2022 (n = 10/63, *p* < 0.001). The current reported grading systems in neurology clerkships mirror the trend in other clerkships as seen in the Association of American Medical Colleges Curriculum reports from 2021 to 2022.^19,20^ According to our 2022 survey, telemedicine has been increasingly incorporated and recognized as an accessible benefit for patients and health care providers.^21^ Survey respondents indicate that medical students perform telemedicine visits in 91% of clerkships, 89% of which are in outpatient clinics exclusively and 2% in both inpatient and outpatient settings. This cannot be compared with previous surveys because this question was not asked previously.

**Table 3:** Grading Scales

	2005^a^ (n = 80)	2012^b^ (n = 95)	2017^c^ (n = 88)	COVID-19 surge 2020–2021^d^ (n = 62)	2022^e^ (n = 63)
H, HP, P, F (%)	56.3	54.7	62.5	45.2	63.5
H, P, F (%)	16.3	18.9	10.2	4.8	11.1
P, F (%)	5.0	2.1	5.7	41.9^a,b,c,e^	15.9^b^
A, B, C, D, F (%)	11.3	7.4	9.1	4.8	6.3
Other (%)	11.3	16.8	12.5	3.2	3.2

F = fail; H = honors; HP = high pass; P = pass.

Superscript letters indicate the Pearson χ^2^ test (*p* < 0.001).

### Postclerkship Experiences

A higher percentage of respondents are offering neurology subinternships in 2022 (89%, n = 65) than in 2012 (71%, n = 97) (*p* = 0.046). In addition, most respondents offer multiple neurology elective options including subspecialty neurology (66%), neurointensive care (60%), ambulatory neurology (58%), and neurology research (52%). As noted in previous years, most respondents (80%) require a neurology core clerkship as a pre-requisite for a neurology elective. While there was an increase in the percentage of students taking a neurology elective between 2005 and 2017 (30% of respondents in 2017 reported that >10% of students completed a neurology elective, compared with 13% of respondents in 2005, *p* = 0.04), the number is unchanged in the 2022 survey at 27%. 87% of respondents report between 2 and 9 students applying to a neurology residency program every year, which has remained steady over the past 5 years. Although the number of respondents reporting >9 students applying to a neurology residency program per year has numerically increased to 7.7% in 2022 (n = 5/65) from 3.3% in 2017 (n = 3/90) and 1% in both 2012 (n = 1/96) and 2005 (n = 1/79), valid statistical comparisons could not be performed because of a low number. Among respondents, those who reported more than 6 medical students per year applying to a neurology residency had a neurology clerkship in the third year (34% of respondents). By contrast, only 11% of respondents who reported more than 6 medical students applying to a neurology residency had a neurology clerkship in the fourth year, third or fourth year, or other years, but this difference was not significant (*p* = 0.106).

### Faculty Teaching in the Neurology Clerkship

Similar to previous years, respondents report that students rotating on the neurology clerkship are supervised by full-time faculty (95%). 94% of clerkship students are also taught by residents and fellows. Salaried part-time or voluntary clinical faculty continue to be involved in clerkship student education (45% in 2022 [n = 29/64], 48% in 2017 [n = 43/89], 68% in 2012 [n = 66/97], and 47% in 2005 [n = 38/81], where the proportion in 2012 was higher than in all 3 other years) (*p* = 0.006). The proportion of nonacademic community practitioners teaching students has remained unchanged over the past 17 years (41% in 2022 [n = 26/64], 42% in 2017 [n = 37/89], 29% in 2012 [n = 28/97], and 42% in 2005 [n = 34/81]) (*p* = 0.199). The proportion of medical students taught by advanced practice providers is similar in 2017 (34%, n = 30/89) to 2022 (39%, n = 25/64) (*p* = 0.496). Respondents report that 23% of students per year are based at off-campus community sites as their main or primary clinical exposure. 42% of respondents' institutions provide compensation or benefits to the community faculty involved in clerkship teaching, largely through giving adjunct or voluntary faculty status (91%) and/or access to neurology education opportunities (82%). There is a lower proportion of respondents who list stipend or salary support as a compensation type provided in 2022 (27%, n = 3/11) compared with 2017 (71%, n = 10/14), but a statistical difference could not be calculated because of low number. Most respondents' institutions do not provide salary support or supplemental honorarium to academic faculty teaching medical students in the neurology clerkship (80%), apart from the CD.

From 2005 to 2022, there has been significant increase in recognition for respondents' faculty teaching efforts in the promotion process (*p* = 0.014, Table 4). The types of education-related faculty development activities available to the neurology clerkship leadership include local faculty development workshops (81%), national faculty development workshops (38%), faculty mentoring (28%), and degree programs (17%). Use of online offerings for faculty development educational courses increased in 2022 (34%, n = 18/53) from 2017 (14%, n = 11/80) (*p* = 0.006). Financial support for the CD to attend the AAN Annual Meeting remained similar in 2022 at 45% (n = 24/53) to that in 2017 (47%, n = 38/80) (*p* = 0.802).

**Table 4:** Importance of Teaching in Promotion

	2005 (n = 78)	2012 (n = 96)	2017 (n = 89)	2022 (n = 65)
Not at all (%)	12.8	5.2	7.9	4.6
Somewhat important (%)	71.8	62.5	49.4	35.4
Very important (%)	15.4	25.0	24.7	38.5
Extremely important (%)	0.0	7.3	18.0	21.5

### Departmental and Institutional Support for the Neurology Clerkship

Respondents report mean protected time, or full-time equivalent (FTE), for their efforts as the neurology CD at 0.27 FTE (SD = 0.14, n = 62), which has risen compared with 0.20 FTE in 2012 (SD = 0.14, n = 96) (*p* = 0.016). The 2017 value of 0.24 FTE was not different from the value in either year (*p* > 0.05). CD respondents perceive that the mean FTE support should be 0.39 FTE in 2022 compared with 0.32 FTE in 2012 (*p* = 0.018), with no difference compared with 2017 (0.35 FTE). Over the past 17 years, support for CDs receiving protected time between 0.26 and 0.5 FTE has numerically increased to 42% in 2022 from 31% in 2017, 19% in 2012, and 6.5% in 2005 (χ^2^ invalid due to low number). In the 2022 survey, most of the respondents have an assistant, associate, or co-director for the neurology clerkship (71%), similar to 57% in 2017 (*p* = 0.078). The mean protected time for the assistant, associate, or co-director dedicated to the clerkship is 0.15 FTE and was similar to 2017 at 0.17 FTE (*p* > 0.05). The mean administrative support for coordinators for the neurology clerkship was 0.43 FTE in 2017 and was similar to 0.52 FTE in 2022 (*p* > 0.05). Support for coordinators receiving between 0.76 FTE and 1.0 FTE for the clerkship has increased from 7.8% in 2005, 14.6% in 2012, and 18.8% in 2017 to 33.9% in 2022 (*p* < 0.001) (Table 1).

### CD Profile

Among those who responded to the survey, 54% are female. Female respondents are similar in age to male respondents (48 years, *p* > 0.05). Both male and female respondents have been in the role for an average of 8 years. The proportion of CDs who have been in the role for over 12 years has remained unchanged from 8.5% in 2005, 15.6% in 2012, 23.3% in 2017, and 18.8% in 2022 (*p* > 0.05). The academic rank of survey respondents varies widely (Table 1). Most use a clinical educator promotion track (58%); however, a considerable proportion (14%) report that this is not an option at their institution. The proportion of CDs in a clinical educator track (58%) is the same as in 2017 and varied from 2012 (48%, *p* = 0.593). CD respondents often serve in additional administrative roles, including hospital/clinic administration (28%); neurology division chief/director (23%); fellowship director (21%); and others (>30%) such as department chair, vice chair, associate program or fellowship director, and education dean at their medical school. 7% of CD survey respondents are also the residency program directors. Most are involved with medical school and departmental education committees (76%) and in formal and informal mentorship programs outside clerkship responsibilities (81%).

Among respondents, over half of CDs are involved in medical education research (55%) and state their needs in developing their medical education research career as learning to measure outcomes, networking and developing collaborations with other programs, learning statistical methods in education research and design, and learning educational concepts. When asked about the importance of teaching in the promotion process at their medical school, the proportion of respondents rating it as “extremely important/essential” has statistically increased from 7% in 2012 and 18% in 2017 to 22% in 2022 (*p* = 0.014). Most CD respondents (87%) indicate they are very or somewhat satisfied. Most report some level of burnout in their role (60%), and 16% indicate frequent burnout. 33% of CDs have considered relinquishing their role in the next 12 months. For those who have considered relinquishing their role, the top 4 reasons are too many competing obligations (55%), lack of sufficient protected time (50%), “hassle factor” of being a CD (45%), and transitioning the role to a junior faculty mentee (45%). CDs note that other reasons such as lack support from leadership, opportunities for other leadership roles, and learning environment changes may also influence their decision to leave their role.

## Discussion

Neurology education continues to play an important role in medical schools across the United States, and neurology clerkships have demonstrated resilience through the COVID-19 surge by continued consistency in the overall structure, administration, and curriculum throughout the United States. With most of the respondents reporting similar findings to prepandemic surveys, CDs were able to successfully navigate the pandemic and reimplement their curriculum. While the number of reported required neurology clerkships has not changed over the past 17 years, the timing of the clerkship has shifted earlier. With new models of curriculum delivery, the proportion of clerkships taken exclusively in the third year has hovered just around 50% while those taken only in the fourth year have decreased. The earlier exposure to neurology is an encouraging finding because fourth-year clerkships may occur too late in training for students to choose neurology as a career. Previous work has shown that early clinical and foundational science exposure to neurology and neurosciences is crucial to students' decisions to pursue neurology.^22^ We recognize there are limitations within this study. Although the survey received a 47% response rate (n = 72 of 152 CDs), caution should be used when generalizing to all CDs. A lower response rate is noted compared with previous survey years despite our best efforts to reach a diverse and representative sample. The results raise concerns about the potential for nonresponse bias and may affect the generalizability of the findings.

The COVID-19 surge caused changes to the neurology clerkship organization and curriculum. There was a predictable reduction in in-person learning and an increase in “Pass, Fail” only grading during the initial COVID-19 surge. Since that time, however, clerkship structure and grading have generally reverted back to 2017 pre–COVID-19 state, with the exception of more clerkships using “Pass, Fail” grading. It is unclear whether this trend will continue or whether the distribution of grading systems used will continue to revert to the 2017 stratification with more time. Outpatient telemedicine visits are being used by most respondents surveyed, and curricula have been developed to teach learners how to perform telehealth visits.^23^ This change was an immediate response to isolation restraints posed by COVID-19, and it will be interesting to see whether telemedicine remains a large part of the student experience in future years.^24,25^ The time spent in online learning nearly doubled during the COVID-19 surge, and a number of online clerkship curricula have been developed in various specialties.^26–29^ Although in-person learning time has reverted to pre–COVID-19 numbers, it remains to be seen whether CDs will also keep the various online learning strategies brought about during the pandemic.

It is evident that there is greater recognition for clinician educators in medical schools than in previous years. Teaching is increasing in importance and is increasingly used as a distinction for promotion. FTE support has increased for CDs and administrative support staff. CDs still report feeling undersupported by the amount of FTE funded for their role. Mean support remains low even as the number of medical schools that require the neurology clerkship increases in the United States. The increasing regulatory requirements and administrative responsibilities have been recognized by others as noted in the 2017 national survey of internal medicine CDs.^30^ Curricular innovations are accelerating in undergraduate medical education, but faculty often lack support and resources to bring such innovations to their own clerkships.^31^ The AAN provides a published neurology curriculum and educational resources through quarterly CNCD meetings and various online resources.

Neurology CDs frequently report experiencing burnout for a variety of reasons, including lack of protected time and competing obligations. The Alliance for Clinical Education has recognized the growing complexity of clerkships across the nation and updated their 2003 recommendations in 2021 to include explicit recommendations to provide a minimum of 0.5 FTE for the role of CD.^32^ Many of the reasons for burnout listed by CDs directly relate to the “hassle factor,” where the level of inconvenience associated with the role influences satisfaction as a neurology CD. School and department leaders can provide direct salary support and protected time and offer support through various educational opportunities including faculty development programs, attendance for the AAN annual meeting, and medical education degree programs.

Trends in neurology undergraduate education have included a migration of the clerkship away from the fourth year toward the third and even the second year, sometimes in a longitudinal clerkship format. Despite the COVID-19 pandemic “surge,” much of the neurology clerkship structure in the United States has remained stable over the past 17 years since the first CNCD survey in 2005. Many respondents remain unaware of the CNCD-published neurology curriculum, which may be a helpful tool in supporting their role. CDs remain committed to their position and have maintained a supportive educational experience in the form of neurology clerkships, subinternships, and electives. They report being more satisfied in their role than ever yet still experience stressors such as lack of protected time that highlight the need for continued efforts to support and promote those educating our future physicians.

The AAN could explore ways to reduce the “hassle” factor of being a CD and continue to advocate for protected time for CDs and their support staff. As medical education becomes more important to the promotion process, the AAN could develop resources to support their members in teaching roles and continue to promote the CNCD-published neurology curriculum.

## Appendix Authors

**Table:**

Name	Location	Contribution
Stephen VanHaerents, MD	Department of Neurology Feinberg School of Medicine, Northwestern University, Chicago, IL	Drafting/revision of the manuscript for content, including medical writing for content; major role in the acquisition of data; study concept or design; analysis or interpretation of data
Doris Kung, DO	Baylor College of Medicine, Houston, TX	Drafting/revision of the manuscript for content, including medical writing for content; major role in the acquisition of data; study concept or design; analysis or interpretation of data
Erin Furr- Stimming, MD	University of Texas Health Science Center at Houston, McGovern Medical School, Houston, TX	Drafting/revision of the manuscript for content, including medical writing for content; major role in the acquisition of data; study concept or design; analysis or interpretation of data
Jeremy K. Cutsforth-Gregory	Mayo Clinic, Rochester, MN	Drafting/revision of the manuscript for content, including medical writing for content; major role in the acquisition of data; study concept or design; analysis or interpretation of data
Joshua Weaver, MD	Weill Cornell Medical College, New York City, NY	Drafting/revision of the manuscript for content, including medical writing for content; major role in the acquisition of data; study concept or design
Carolyn M. Cahill	American Academy of Neurology, Minneapolis, MN	Major role in the acquisition of data; analysis or interpretation of data
Tasha Ostendorf	American Academy of Neurology, Minneapolis, MN	Major role in the acquisition of data; analysis or interpretation of data
Christopher M. Keran	American Academy of Neurology, Minneapolis, MN	Drafting/revision of the manuscript for content, including medical writing for content; analysis or interpretation of data

## Glossary

AAN: American Academy of Neurology
CD: clerkship director
CNCD: Consortium of Neurology Clerkship Directors
COVID-19: coronavirus disease 2019
FTE: full-time equivalent
IRB: institutional review board
LP: lumbar puncture

## References

[1] Albert DV, Yin H, Amidei C, Dixit KS, Brorson JR, Lukas RV. Structure of neuroscience clerkships in medical schools and matching in neuromedicine. Neurology. 2015;85(2):172-176. doi:10.1212/WNL.0000000000001731 26070342

[2] Dall TM, Storm MV, Chakrabarti R, et al. Supply and demand analysis of the current and future US neurology workforce. Neurology. 2013;81(5):470-478. doi:10.1212/WNL.0b013e318294b1cf 23596071 PMC3776531

[3] Safdieh JE, Quick AD, Korb PJ, et al. A dozen years of evolution of neurology clerkships in the United States: looking up. Neurology. 2018;91(15):e1440-e1447. doi:10.1212/WNL.0000000000006170 30194245

[4] Simon RP, Aminoff MJ, Greenberg DA. Clinical Neurology. 4th ed. Appleton & Lange; 1999.

[5] Toy Eugene C., Mancias Pedro, Furr-Stimming Erin, Kung Doris. Case Files: Neurology. 2nd ed. McGraw-Hill Medical; 2013.

[6] Drislane F. Blueprints Neurology. 3rd ed. Wolters Kluwer Health/Lippincott William & Wilkins; 2009.

[7] Gelb DJ. Introduction to Clinical Neurology. 5th ed. Oxford University Press; 2016.

[8] Kaplan T, Milligan T. Introduction. In: The Neurology Video Primer (online edition). Oxford Academic; 2018.

[9] Ropper Allan, Samuels Martin, Klein Joshua P., Prasad Sashank. Adams and Victor’s Principles of Neurology. 12th ed. McGraw-Hill Education LLC; 2023.

[10] Anschel D, DiCapua D. Neurology: PreTest Self-Assessment and Review. 10th ed. McGraw Hill; 2021.

[11] Berkowitz A, editor. Clinical Neurology & Neuroanatomy: A Localization-Based Approach. 2nd ed. McGraw-Hill Education; 2022.

[12] Blumenfeld H Neuroanatomy through Clinical Cases. 3rd ed. Sinauer Associates, an imprint of Oxford University Press; 2022.

[13] Hauser Stephen, Josephson Scott. Harrison’s Neurology in Clinical Medicine. 2nd ed. McGraw-Hill Medical; 2010.

[14] Johnson P. Raven Neurology Review: Clinical Neurology for Medical Students. CreateSpace Independent Publishing Platform; 2018.

[15] Louis Elan D., Mayer Stephan A., Rowland Lewis P. Merritt’s Neurology. 13th ed. Wolters Kluwer Health; 2016.

[16] Biller J. Practical Neurology. 3rd ed. Wolters Kluwer Health/Lippincott Williams & Wilkins; 2009.

[17] Cucchiara BL, Price RS. Decision-Making in Adult Neurology. Elsevier; 2021.

[18] Hauer KE, Lucey CR. Core clerkship grading: the illusion of objectivity. Acad Med. 2019;94(4):469-472. doi:10.1097/ACM.0000000000002413 30113359

[19] Association of American Medical Colleges. Number of Medical Schools Using Selected Grading Systems in Required Clinical Clerkships. AAMC Curriculum Inventory, 2018-2022. Accessed August 2024. https://www.aamc.org/data-reports/curriculum-reports/data/grading-systems-used-medical-school-programs

[20] Association of American Medical Colleges. Methods Used for Clinical Knowledge and/or Skills Assessment in Clinical Clerkship Experiences in 2021-2022, by Topic Area. Liaison Committee on Medical Education (LCME) Annual Questionnaire Part II, 2021-2022. Accessed August 2024. https://www.aamc.org/data-reports/curriculum-reports/data/clerkship-assessment-methods-clinical-knowledge-and-skills

[21] Shaver J. The state of telehealth before and after the COVID-19 pandemic. Prim Care. 2022;49(4):517-530. doi:10.1016/j.pop.2022.04.002 36357058 PMC9035352

[22] Jordan JT, Cahill C, Ostendorf T, et al. Attracting neurology's next generation: a qualitative study of specialty choice and perceptions. Neurology. 2020;95(8):e1080-e1090. doi:10.1212/WNL.0000000000009461 32332127

[23] Han SC, Stainman RS, Busis NA, Grossman SN, Thawani SP, Kurzweil AM. Curriculum innovations: a comprehensive teleneurology curriculum for neurology trainees. Neurol Educ. 2023;2(3):e200084. doi:10.1212/NE9.0000000000200084 39359705 PMC11419297

[24] Abraham HN, Opara IN, Dwaihy RL, et al. Engaging third-year medical students on their internal medicine clerkship in telehealth during COVID-19. Cureus. 2020;12(6):e8791. doi:10.7759/cureus.8791 32724740 PMC7381847

[25] Henschen BL, Jasti H, Kisielewski M, Pincavage AT, Levine D. Teaching telemedicine in the COVID-19 era: a national survey of internal medicine clerkship directors. J Gen Intern Med. 2021;36(11):3497-3502. doi:10.1007/s11606-021-07061-4 34494207 PMC8423074

[26] Mason MW, Aruma JFC. An orthopaedic virtual clinical clerkship for visiting medical students: early successes and future implications. J Surg Educ. 2022;79(2):535-542. doi:10.1016/j.jsurg.2021.09.019 34666935

[27] Redinger KE, Greene JD. Virtual emergency medicine clerkship curriculum during the COVID-19 pandemic: development, application, and outcomes. West J Emerg Med. 2021;22(3):792-798. doi:10.5811/westjem.2021.2.48430 34125062 PMC8202996

[28] Shin TH, Klingler M, Han A, et al. Efficacy of virtual case-based general surgery clerkship curriculum during COVID-19 distancing. Med Sci Educ. 2021;31(1):101-108. doi:10.1007/s40670-020-01126-5 33200037 PMC7654350

[29] Sukumar S, Zakaria A, Lai CJ, Sakumoto M, Khanna R, Choi N. Designing and implementing a novel virtual rounds curriculum for medical students' internal medicine clerkship during the COVID-19 pandemic. MedEdPORTAL. 2021;17:11106. doi:10.15766/mep_2374-8265.11106 33768143 PMC7970635

[30] Glod SA, Alexandraki I, Jasti H, et al. Clerkship roles and responsibilities in a rapidly changing landscape: a national survey of internal medicine clerkship directors. J Gen Intern Med. 2020;35(5):1375-1381. doi:10.1007/s11606-019-05610-6 31898141 PMC7210333

[31] Blood A, Farnan J, Fitz-William W. Curriculum changes and trends 2010-2020: a focused national review using the AAMC curriculum inventory and the LCME annual medical school questionnaire Part II. Acad Med. 2020;95(9S):S5-S14. doi:10.1097/ACM.0000000000003484 33626633

[32] Morgenstern BZ, Roman BJB, DeWaay D, et al. Expectations of and for clerkship directors 2.0: a collaborative statement from the alliance for clinical education. Teach Learn Med. 2021;33(4):343-354. doi:10.1080/10401334.2021.1929997 34294018

